# Supplementary material for: Patterns of Intron Gain and Loss in Fungi
Source: PLoS Biol. 2004 Nov 30;2(12):e422. doi: 10.1371/journal.pbio.0020422 (PMC532390; doi:10.1371/journal.pbio.0020422)
Supplement: Table S1 — Also available at http://genes.mit.edu/NielsenEtAl/. (4.3 MB ZIP). [file pbio.0020422.st001.zip › NielsenEtAl/html/1364.html]

AN2153.1.NCU08026.1.MG07084.1.FG04277.1


```
 CLUSTAL W (1.82) Multiple Sequence Alignments - Introns Inserted


Sequence 1: NCU08026.1	286 aa
Sequence 2: MG07084.1	302 aa
Sequence 3: FG04277.1	283 aa
Sequence 4: AN2153.1	324 aa
Alignment Length: 325 aa
Number Identitical Residues: 123 aa
Alignment Score (without introns) 6299


MG07084.1 	MTDRRRINGPVGTTLPPVFFDEPIP---DKPGPR-TRSRPPNAIRKIF1LKTGITPSASG
NCU08026.1	MADRRRINGPVGTTNPPIYDDAPEK---QSEGFKVTRSRPANVIRKMY1LKTGVTPSASG
FG04277.1 	MTDRRRINGPGGNTVPPVFEAD------ESIVPR--RTRAANGIRAQY1LKTGVTPSASG
AN2153.1  	MTDRRRINGPPGGTRPPIFASSIKPTATATAAERPQRQRQPNELRKIF1LKTGLIPSASG
          	*:******** * * **::      ::: .   :  * * .* :*  : ****: *****

MG07084.1 	SAYLEIENPVVSGKEGET------STSGLKLSCTVHGPRALPRSTPFSPHIILSTHIKYA
NCU08026.1	SAYLEIES---SGNGG----------SGLKLSCSVHGPRALPRSTPFSPHIILSTHVKFA
FG04277.1 	SAYLEIESQDASGSKG------------MKLTCTVHGPRSLPRSAPFSPHMVLSTHVKYA
AN2153.1  	SSYLEFEPSASLSAARASPKFITPPSSSLKLACTVHGPKPLPRSATFSPNLVLTTHVKYA
          	*:***:*     .    :..  :..::.:**:*:****:.****:.***:::*:**:*:*

MG07084.1 	PFATKQRRGYIRDSTERDLSVHLETALRGVIIADRWPKSGVDVIVSVIEGDQDRRLPLQD
NCU08026.1	PFATKQRRGYIRDPIERDLGTHLETALRGSIIADRWPKSGVDIIISIVEGEQDR---ETS
FG04277.1 	PFATRQRRGYLRDSSERDLSSHLEAALRGAIIADRWPKSGVDVIVTVIEGDQAR---EVA
AN2153.1  	PFAARKRKGHIRDASERDLGVHLETALRGVIVAERWPKSGLDITITILEAEDDR-WWGDA
          	***:::*:*::**. ****. ***:**** *:*:******:*: ::::*.:: *      

MG07084.1 	SGSGEEAWSTMSVLSGCITVASAALADAGIDCVDMVAGGVAALVRDDSDGAKSVEGGSIV
NCU08026.1	KAQGIEAWDTMNALSGCITVASAALADAGIDCVDTVAGGVAALVQDAGNDAAP----SIV
FG04277.1 	VEQGSEEWDMMNVLSGCITVAAAALADAGIDCVDTVSGGVAALVLGQ-DNNEP----IMV
AN2153.1  	PDSHDAAWGMMNVLAGCITAASAAIADARIDCLDLVAGGVAAVVVDKLADGNGNSCARLM
          	  .    *. *..*:****.*:**:*** ***:* *:*****:* .   .    . . ::

MG07084.1 	VDPVPAEHDKIVAACCVAYLPNRDEVTNLWFKGDAA-----ASDSAL-YLNIVNNSVRAS
NCU08026.1	VDPIPSEHEKVLAACCIAYLPTRQEVTNLWFKGDLP-----ASDMDV-YTQLIEKGIQAS
FG04277.1 	LDPVPSEHSQILAACCVAYLPERDEITNLWLKGSLP-----SSDANL-HRGLVARAVHAS
AN2153.1  	LDTDPAEHQSILSACVVAYMPGRDEITELWLKGDNSKAAVGTTDQNLSHDALIDGAVVAA
          	:*. *:**..:::** :**:* *:*:*:**:**. ..:: .::*  :::  ::  .: *:

MG07084.1 	KIANRVLVDSLKETLQQQD-------
NCU08026.1	KSANRVLVDCLNETVG----------
FG04277.1 	KGANLAVITSLNESILAV--------
AN2153.1  	RGAHSVLAEAVRESAMRFAGQSSGSS
          	: *: .:  .:.*:     ..::.::
```
